# Complement Receptor 3 Has Negative Impact on Tumor Surveillance through Suppression of Natural Killer Cell Function

**DOI:** 10.3389/fimmu.2017.01602

**Published:** 2017-11-20

**Authors:** Cheng-Fei Liu, Xiao-Yun Min, Naiyin Wang, Jia-Xing Wang, Ning Ma, Xia Dong, Bing Zhang, Weiju Wu, Zong-Fang Li, Wuding Zhou, Ke Li

**Affiliations:** ^1^Core Research Laboratory, The Second Affiliated Hospital, School of Medicine, Xi’an Jiaotong University, Xi’an, China; ^2^Medical Research Council (MRC) Centre for Transplantation, King’s College London, Guy’s Hospital, London, United Kingdom; ^3^National Local Joint Engineering Research Centre of Biodiagnostics and Biotherapy, The Second Affiliated Hospital, School of Medicine, Xi’an Jiaotong University, Xi’an, China; ^4^Shaanxi Provincial Clinical Research Center for Hepatic & Splenic Diseases, Xi’an, China

**Keywords:** complement receptor 3, Melanoma, iC3b, natural killer cell function, tumor surveillance

## Abstract

Complement receptor 3 (CR3) is expressed abundantly on natural killer (NK) cells; however, whether it plays roles in NK cell-dependent tumor surveillance is largely unknown. Here, we show that CR3 is an important negative regulator of NK cell function, which has negative impact on tumor surveillance. Mice deficient in CR3 (CD11b^−/−^ mice) exhibited a more activated NK phenotype and had enhanced NK-dependent tumor killing. In a B16-luc melanoma-induced lung tumor growth and metastasis model, mice deficient in CR3 had reduced tumor growth and metastases, compared with WT mice. In addition, adaptive transfer of NK cells lacking CR3 (into NK-deficient mice) mediated more efficient suppression of tumor growth and metastases, compared with the transfer of CR3 sufficient NK cells, suggesting that CR3 can impair tumor surveillance through suppression of NK cell function. *In vitro* analyses showed that engagement of CR3 with iC3b (classical CR3 ligand) on NK cells negatively regulated NK cell activity and effector functions (i.e. direct tumor cell killing, antibody-dependent NK-mediated tumor killing). Cell signaling analyses showed that iC3b stimulation caused activation of Src homology 2 domain-containing inositol-5-phosphatase-1 (SHIP-1) and JNK, and suppression of ERK in NK cells, supporting that iC3b mediates negative regulation of NK cell function through its effects on SHIP-1, JNK, and ERK signal transduction pathways. Thus, our findings demonstrate a previously unknown role for CR3 in dysregulation of NK-dependent tumor surveillance and suggest that the iC3b/CR3 signaling is a critical negative regulator of NK cell function and may represent a new target for preserving NK cell function in cancer patients and improving NK cell-based therapy.

## Introduction

Natural killer (NK) cells comprise 2–13% of all circulating lymphocytes and mediate spontaneous killing of tumor, foreign cells, and aberrant host cells by exocytosis of granzyme- and perforin-containing cytoplasmic granules. They also produce cytokines (e.g., IFN-γ) to regulate other immune cell functions (1, 2). NK cells express an array of receptors including the activating receptors (e.g., NKp46) and inhibitory receptors [e.g., CD158b (in man), ly49I/C (in mice)] and cytokine/chemokine receptors (3). Through these receptors, NK cells can sense and respond to many distinct stimuli and undergo functional modulation participating in immune responses under both physiological and pathological conditions (4, 5).

In the context of malignancy, both clinical and experimental studies have provided substantial evidence suggesting that NK cells are fundamental to elimination of tumor cells and the control of tumor metastasis (6, 7). It has been shown that NK cells can eliminate tumor cells from the circulation, prevent the dissemination of metastatic tumors, and suppress tumor outgrowth in experimental models (8–10). In the clinic, tumor infiltration by NK cells is associated with a better prognosis in several types of carcinomas (11–14). In hematopoietic stem-cell transplantation (HSCT) and adoptive NK cell immunotherapy, NK cells are critical for controlling and/or eradicating high-risk leukemia (15–17). In anti-tumor antibody therapy, NK-mediated killing is required for curative efficacy of antibody. Together, these observations provide compelling evidence that NK cells play a significant role in tumor surveillance and can be used in therapeutic settings against cancer. However, tumor cell-induced excessive inflammatory responses can promote the development of immunosuppressive microenvironment leading to compromised NK cell function which enhances tumor growth and metastasis (18–20); current NK cell-based therapy also faces a lot of challenges, e.g., functional decline of the reconstituted NK cells in acute myeloid leukemia patients following HSCT and rapid loss of cytotoxic activity of infused NK cells that have been expanded *ex vivo* (21, 22). The factors that could dysregulate NK cell function in both situations are not clear. More thorough understanding of mechanisms that regulate NK cell function and identifying the mediators that lead to NK dysfunction are required for improvement of NK-based therapy.

The complement system is an integral part of innate immunity (23). Spontaneous and well-controlled complement activation occurs under physiological conditions. Increased complement activation takes place in response to infection and to a diverse set of innate molecules and signatures, particularly under pathological conditions. Once activated, the complement cascade generates a set of effector molecules, including the large fragment C3b and its further degraded products iC3b and C3d, the small fragments (C3a and C5a) and the terminal product C5b-9. Apart from mediating a direct killing of foreign cell/pathogens by C5b-9, activation of complement also plays important roles in immune regulation through engagement of complement receptors (e.g., C3aR, C5aR, CR1, CR2, and CR3) on immune cells with respective complement cleavage products (e.g., C3a, C5a, C3b, C3d, and iC3b) (23–26).

Complement receptor 3 [also known as Mac-1, integrin α(M)β (2), CD11b/CD18] is heterodimeric leukocyte adhesion molecule and abundantly expressed by NK cells both in man and mice. iC3b (inactive product of the cleavage fragment C3b) is the classic ligand for CR3, although non-complement molecules such as ICAM-1 and fibrinogen can also function as a ligand for CR3. iC3b either in fluid phase (with a relative low affinity) or bound to biological surfaces can express biological activities through interaction with CR3 (27, 28). It has been shown that iC3b-CR3 interactions had suppressive effects on antigen-presenting cells and immature dendritic cells, suggesting a negative regulatory role of CR3 in immune cells (29, 30). In terms of tumor, it has been shown that increased soluble iC3b levels were associated with the progression of pancreatic adenocarcinoma, suggesting iC3b as an early biomarker and a potential risk factor for pancreatic carcinoma (31).

Given the abundant expression of CR3 in NK cells, negative regulatory roles of iC3b/CR3 axis in other immune cells and the association of iC3b with tumor progression, we hypothesized that iC3b/CR3 signaling is an important negative regulator of NK cell function, which may have negative impact on tumor surveillance and hinder the efficiency of NK-based and antibody-based therapies. To test the hypothesis, we employed CR3 functional deficient (CD11b^−/−^) mice and several *in vivo* models (i.e., an NK-dependent peritoneal tumor elimination model, a pulmonary B16 melanoma metastases model, and the metastases model combining adaptive transfer of NK cells in NK-deficient mice). We assessed whether CR3-deficient NK cells have enhanced tumor cell killing capacity and whether CR3 deficiency and more specifically CR3-deficient NK cells protect mice from pulmonary metastatic melanoma. We also performed *in vitro* analysis to define the role of CR3 in NK cells. We examined the effects of iC3b-containing serum and iC3b-apoptotic cells on NK cell activation and effector functions using freshly prepared human NK cells. We explored the intracellular signaling pathways responsible for the action of iC3b on NK cell functional regulation. Our results indicate that CR3 signaling negatively regulates NK cell function and impairs NK cell-dependent tumor surveillance in mice.

## Materials and Methods

### Reagents

Normal human serum and C3-depleted serum were purchased from Sigma-Aldrich (Shanghai, China). Cell culture medium and supplements were purchased from Invitrogen China Limited (Beijing, China). Recombinant human IL-2 was purchased from Peprotech China (Suzhou, China). Rituximab was purchased from Roche (Mannheim, Germany). The following fluorochrome-conjugated monoclonal antibodies were used for the flow cytometry detection: FITC-conjugated anti-mouse CD3 (145-2C11), anti-human CD3e *(*SP34), PE-conjugated anti-mouse CD69 (G235-2356), anti*-*human IFN-γ (4S. B3), -Granzyme B (GB11) were purchased from BD Biosciences (Oxford, UK). APC-conjugated anti-mouse NK1.1 (PK136), anti-human CD56 (B159), PE-conjugated anti-mouse NKp46 (29A1.4), CD107a (1D4B), Ly49I/C (YLI-90), and anti-human CD54 (HA58), NKp46 (9E2), CD69 (FN50), CD158b (DX27), CD107a (H4A3), and perforin (dG9) were all purchased from BioLegend (San Diego, CA, USA). Antibody reagents used in signaling pathway studies [i.e., anti-phospho-Src homology 2 domain-containing inositol-5-phosphatase-1 (SHIP1) (Tyr1020), -SAPK/JNK (Thr183/Tyr185), -ERK1/2 (Thr202/Tyr204), -p38 (Thy180/Tyr182), -Akt (Ser473), and anti-SHIP1, -ERK1/2, -JNK, -Akt, and -p38 antibodies] and signaling pathway inhibitors (i.e., ERK inhibitor U0126, JNK inhibitor SP600125) were purchased from Cell Signaling Technology (Danvers, MA, USA). SHIP1 inhibitor (3-alpha-aminocholestane, 3AC) was purchased from Merck (Beijing, China). Mouse monoclonal anti-human iC3b (neo) Ab and the MicroVue iC3b Enzyme Immunoassay kit was from purchased Quidel (San Diego, CA, USA). Rat monoclonal anti-mouse C3b/iC3b/C3c (2/11) was purchased from Hycult Biotech (Uden, the Netherlands). Cytofix/Cytoperm™ Kit with GolgiPlug™ was purchased from BD Biosciences. ToxiLight™ Non-destructive Cytotoxicity BioAssay Kit was purchased from Lonza (Rockland, ME, USA).

### Mice

CD11b^−/−^ mice (Itgam^tm1Rws^) were purchased from Jackson Laboratory (Bar Harbor, ME, USA), E4BP4^−/−^ mice (32) were provided by Professor H. Brady. Both strains have been backcrossed onto C57BL/6 strain for at least 10 generations. C57BL/6 WT mice were purchased from Vital River Laboratories (Beijing, China). Male mice (6–8 weeks old) were used in all experiments; all mice were maintained in specific pathogen-free conditions. The Ethics Review Committee for Animal Experimentation at Xi’an Jiaotong University approved and oversaw all mouse experiments.

### Lung Metastasis Model

Mice [WT or CD11b^−/−^ or NK cell-deficient mice (E4bp4^−/−^ mice)] were inoculated with luciferase-labeled B16 melanoma cells (B16-luc cells, 1 × 10^6^ in 100 µl PBS) by intravenous tail injection at day 0. At day 7 and day 14, mice were shaved, anesthetized, and administered with 30 µg D-luciferin (R&D system, Minneapolis, MN, USA) by intraperitoneal injection. Bioluminescence images were acquired using the IVIS Lumina XRMS Series III (Xenogen, San Francisco, CA, USA). All images were analyzed using Living Image software (Xenogen). Average radiance (p/s/cm^2^/sr) is used to determine the tumor load, which refers to the number of photons (p) per second that are leaving a square centimeter of tissue and radiating into a solid angle of 1 sr. At day 14, mice were sacrificed, the number of tumor nodules on lung surface were also enumerated manually. For adaptive transfer of NK cell experiment, E4bp4^−/−^ mice received 5 × 10^5^ FACS-sorted NK1.1^+^CD3^−^ NK cells prepared from WT or CD11b^−/−^ mice by intravenous tail injection on the day of tumor cell inoculation. The mice received a second dose of NK cells on day 7 following the inoculation.

### NK-Dependent Peritoneal Tumor Elimination Model

RMA-s (NK-sensitive lymphoma cells) and RMA cells (NK non-sensitive lymphoma cells) were incubated with different concentrations of CFSE (5 and 0.5 µM, respectively) for 10 min at 37°C. After wash, RMA-s cells (CFSEhi) (2.5 × 10^6^) and RMA cells CFSElo (1 × 10^6^) were mixed and injected into the peritoneum of WT or CD11b^−/−^ mice. 4 h after tumor injection, peritoneal lavage cells were collected and analyzed by flow cytometry, and the various populations of labeled cells were detected by their differential CFSE fluorescence intensities. To calculate the remaining RMA-s cells, the following formula was used: remaining RMA-s cells = (ratio of recovered cells × the number of injected RMA cells), ratio = (percentage of RMA-s [CFSEhi]/percentage of [CFSElo]).

### Preparation of iC3b (Soluble or Bound)

To generate iC3b containing serum, normal human serum and C3-depleted serum (the control) were treated with zymosan (0.5 mg/ml, Sigma) at 37°C for 40 min, centrifuged to remove insoluble particles. To prepare membrane-bound iC3b, Jurkat T cells were cultured in RPMI-1640 containing 10% FCS until reach plateau-phase, and further cultured in medium containing 2% FCS at 2 × 10^6^ cells/ml for two and half hours, to promote apoptosis. Apoptotic Jurkat cells were then incubated with RPMI-1640 containing 20% normal human serum for 1 h, and the deposition of iC3b on the surface of apoptotic cells was analyzed by flow cytometric analysis. Apoptotic cells incubated with C3-depleted serum having no iC3b deposition served as the control.

### Preparation of Human NK Cells

Human peripheral blood samples were obtained with informed consent from a panel of seven healthy donors (male:female, 4:3) aged between 24 and 41 from the department. The study had been approved by the School Human Studies Ethics Committee. Peripheral blood mononuclear cells (PBMCs) were obtained by Ficoll-Hypaque gradient centrifugation (Lymphoprep, PAA laboratories, Pasching, Austria). Total NK cells were isolated from PBMCs using NK isolation kit (Miltenyi Biotec, Bergisch Gladbach, Germany) according to the manufacturer’s instructions. After the isolation, the purity of the CD56^+^CD3^−^ NK cell preparation was routinely more than 90%. Purified human NK cells were cultured in completed RPMI-1640 (10% heat-inactivated FCS, 50 µM 2-mecaptoethanol, 2 mM glutamine, 50 U/ml penicillin and 50 μg/ml streptomycin) supplemented with 50 pg/ml recombinant human IL-2 at 37°C in 5% CO_2_. In some experiments, iC3b-containing serum (10%) or iC3b-bound apoptotic cells (at 1:1 NK-apoptotic cell ratio) were added into culture medium for indicated time period.

### Phenotypic and Functional Analyses of Human NK Cells by Flow Cytometry

For NK cell activation phenotype, NK cells were stained with a cocktail of directly conjugated antibodies against surface molecules, including anti-CD56 (APC), -CD3e (FITC), -CD54 (PE), -CD69 (PE), -NKp46 (PE), and -CD158b (PE) and followed by flow cytometry. For NK cell function, NK cells were cocultured with K562 cells at 1:1 effector–target (E/T) ratio for 1 h and followed by intracellular staining for CD107a, granzyme B and perforin and followed by flow cytometry. For IFN-γ production, NK cells were cocultured with K562 cells for 2 h, thereafter, BD GolgiStop™ Protein Transport Inhibitor (contains monensin) was added to the coculture and incubation for another 4 h. The cells were then stained intracellularly with anti-IFN-γ antibody and followed by flow cytometry. In some experiments, NK cells were pretreated with specific inhibitor of SHIP-1 (3AC) (33) (0.5 µM), ERK (U0126) (10 µM), and JNK (SP600125) (25 µM), or vehicle controls (culture medium containing 0.002% ethanol for 3AC, and 0.1% DMSO for both U0126 and SP600125) for 16 h. All the experiments were performed using FACS Calibur (Becton Dickinson). Data were analyzed using FlowJo software (Tree Star, Ashland, OR, USA). Mean fluorescence intensity (MFI) and percentage positive cells were determined.

### NK Cytotoxicity Assay

*In vitro* cytotoxicity assay was performed using the ToxiLight™ Non-destructive Cytotoxicity BioAssay Kit (Lonza) following the supplier’s instruction. Briefly, NK cells were treated with bound iC3b for 24 h, and after washing, the NK cells were cocultured with target cells [with NK cell/target cell (E/T) ratios from 20:1 to 1:1] for another 4 h at 37°C. For direct tumor cell killing, K562 cells were used as target cells. For antibody-dependent cell-mediated cytotoxicity (ADCC), Raji cells were used, and Rituximab (5 µg/ml) was present in the coculture. At the end of the incubation, 100 µl of adenylate kinase detection reagent was added to each well, the luminous intensity was detected by SpectraMax^®^ i3 (Molecular Devices, USA).

### Preparation of Murine NK Cells

Murine NK cells were isolated from splenocytes by negative selection using the mouse NK cell isolation kit (Miltenyi Biotec) and further sorted by BD FACS Aria II (Becton Dickinson, San Jose, CA, USA) using anti-NK1.1 and -CD3 antibodies. The purity of sorted NK1.1^+^CD3^−^ cells was consistently more than 95%, as determined by flow cytometry.

### Functional Analysis of Murine NK Cells by Flow Cytometry

2 × 10^5^ purified murine NK cells were cocultured with Yac-1 tumor targets (a 2:1 E/T ratio) for 90 min. The cells were preincubated with mouse anit-CD16-CD32 mAb to block Fc receptors and then with fluorochrome-conjugated anti-NK1.1, -CD3, and -CD107a antibody, followed by flow cytometry. For IFN-γ production, NK cells were incubated with Yac-1 for 1 h, GolgiPlug was added to the cultures, which were incubated for another 4 h. The cells were then stained intracellularly with anti-IFN-γ antibody and followed by flow cytometry.

### Western Blot

Natural killer cells were incubated with bound iC3b and lysed at indicated times. Equal amounts of protein were subjected to SDS-PAGE electrophoresis, and transferred onto PVDF membranes. The membranes were incubated with primary antibody at 4°C overnight, followed by incubation with HRP-conjugated secondary antibody. Protein bands were visualized by Amersham ECL Select™ detection reagent (GE Healthcare Life Sciences, Marlborough, MA, USA).

### Statistical Analysis

Statistical analyses were performed using Graphpad Prism 5.03 software (La Jolla, CA, USA). Unpaired two-tailed Student’s *t*-test was used to compare two groups with unmatched data. Welch’s correction was used when there was heterogeneity of variance. Paired two-tailed Student’s *t*-test was used to compare two groups with matched data. Two-way ANOVA was used to compare the mean values of more than two independent groups. *P* < 0.05 was considered statistically significant.

## Results

### Mice Deficient in CR3 Have a More Activated NK Phenotype and Better NK-Dependent Tumor Killing

**Figure 1:**
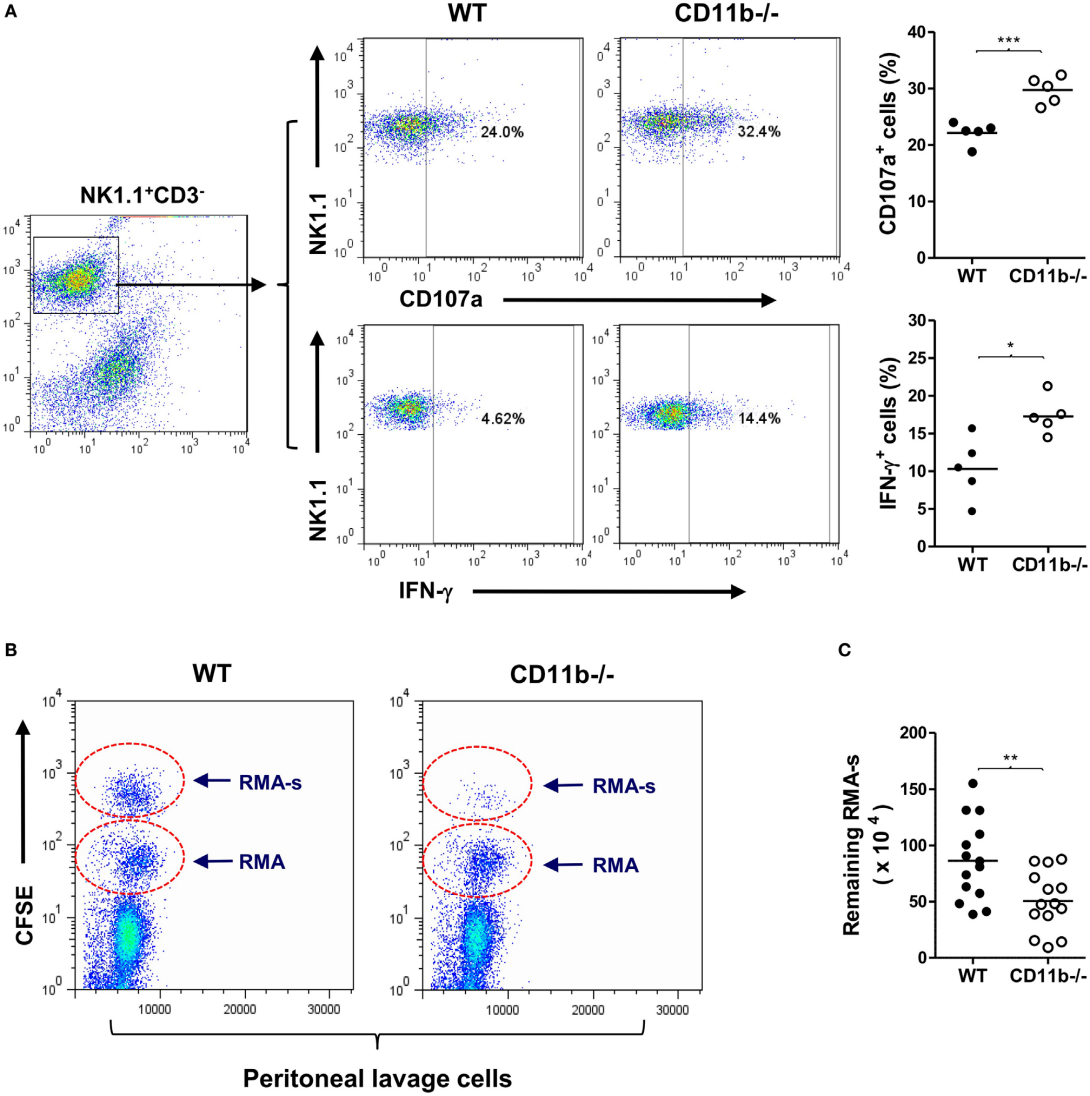
Mice deficient in complement Receptor 3 have a more activated natural killer (NK) phenotype and better NK-dependent tumor killing. **(A)** Expression of CD107a and IFN-γ in purified NK cells of WT and CD11b^−/−^ mice that had been cocultured with Yac-1 tumor cells. Left: representative flow cytometry dot plots showing percentage of CD107a^+^ or IFN-γ^+^ cells in gated NK1.1^+^CD3^−^ cells. Right: quantification of percentage of CD107a^+^ or IFN-γ^+^ cells obtained from five mice per group, performed in five individual experiments. **(B)** Representative peritoneal lavage cell profiles of WT and CD11b^−/−^ mice following intraperitoneal injection of tumor cells. **(C)** Quantification of remaining RMA-s cells from WT and CD11b^−/−^ mice (*n* = 14, pooled from four individual experiments). Data in **(A,C)** were analyzed by unpaired two-tailed Student’s *t*-test, *P < 0.05, **P < 0.01, comparing CD11b^−/−^ and WT mice. Each dot represents an individual mouse; small horizontal lines indicate the mean.

CD11b is the α-subunit of CR3, therefore CD11b^−/−^ mice cannot form functional CR3, and hence the interaction of CR3 with ligand does not occur. It has been reported that NK cells from CD11b^−/−^ mice displayed a more activated peripheral NK cell phenotype and had implications in liver disease (32). However, whether the CD11b^−/−^ NK cell phenotype is relevant to malignancy is unknown. To address this, we first examined NK cell maturation state in CD11b^−/−^ and WT mice. There were similar percentages of NK1.1^+^CD3^−^ (total NK cells) or NK1.1^+^CD3^-^DX5^+^ (matured NK cells) of splenic cells and bone marrow cells for CD11b^−/−^ and WT mice, suggesting that CD11b^−/−^ mice had a no alteration of NK cell maturation (Figure S1 in Supplementary Material). We also confirmed that peripheral NK cells lacking CR3 (from CD11b^−/−^ mice) exhibited increased expression of activating receptors/markers (NKp46 and CD69) and decreased expression of inhibitory receptor Ly49I/C, compared with CR3 sufficient NK cells (from WT mice) (Figure S2 in Supplementary Material). Furthermore, we showed that NK cells lacking CR3 exhibited increased NK cell functional activity after exposure to tumor cells (measured by expression of CD107a and IFN-γ), compared with CR3 sufficient NK cells (Figure 1A).

Next, we assessed whether lack of CR3 influences NK cytotoxicity *in vivo* using an NK-dependent lymphoma rejection model (34). We initially confirmed that peritoneal NK cells of CD11b^−/−^ mice exhibited a more activated phenotype than that of WT mice (Figure S3 in Supplementary Material), and there was a large amount of deposition of activated C3 (C3b/iC3b) on tumor cells after 1 h of being injected into the peritoneal cavity (Figure S4 in Supplementary Material), indicating that sufficient ligands of CR3 can be generated rapidly. We then administered a mixture of NK-sensitive lymphoma cells (RMA-s) and NK-non-sensitive lymphoma cells (RMA) in WT and CD11b^−/−^ mice by intraperitoneal injection, the RMA cells being used as an internal control to normalize the remaining RMA-s cells. Four hours after the injection, we collected peritoneal lavage cells, performed flow cytometry, and calculated the remaining RMA-s cells. There were significantly fewer RMA-s tumor cells remained in CD11b^−/−^ mice than that in WT mice, indicating that NK-dependent tumor cell killing is more efficient in CD11b^−/−^ mice than that in WT mice (Figures 1B,C). These results demonstrate that lack of CR3 leads to enhanced NK activity and effector function in tumor elimination, supporting a role for CR3 in NK cell functional activity suppression.

### CR3-Dependent Suppression of NK Cell Function Has Negative Impact on Tumor Surveillance

**Figure 2:**
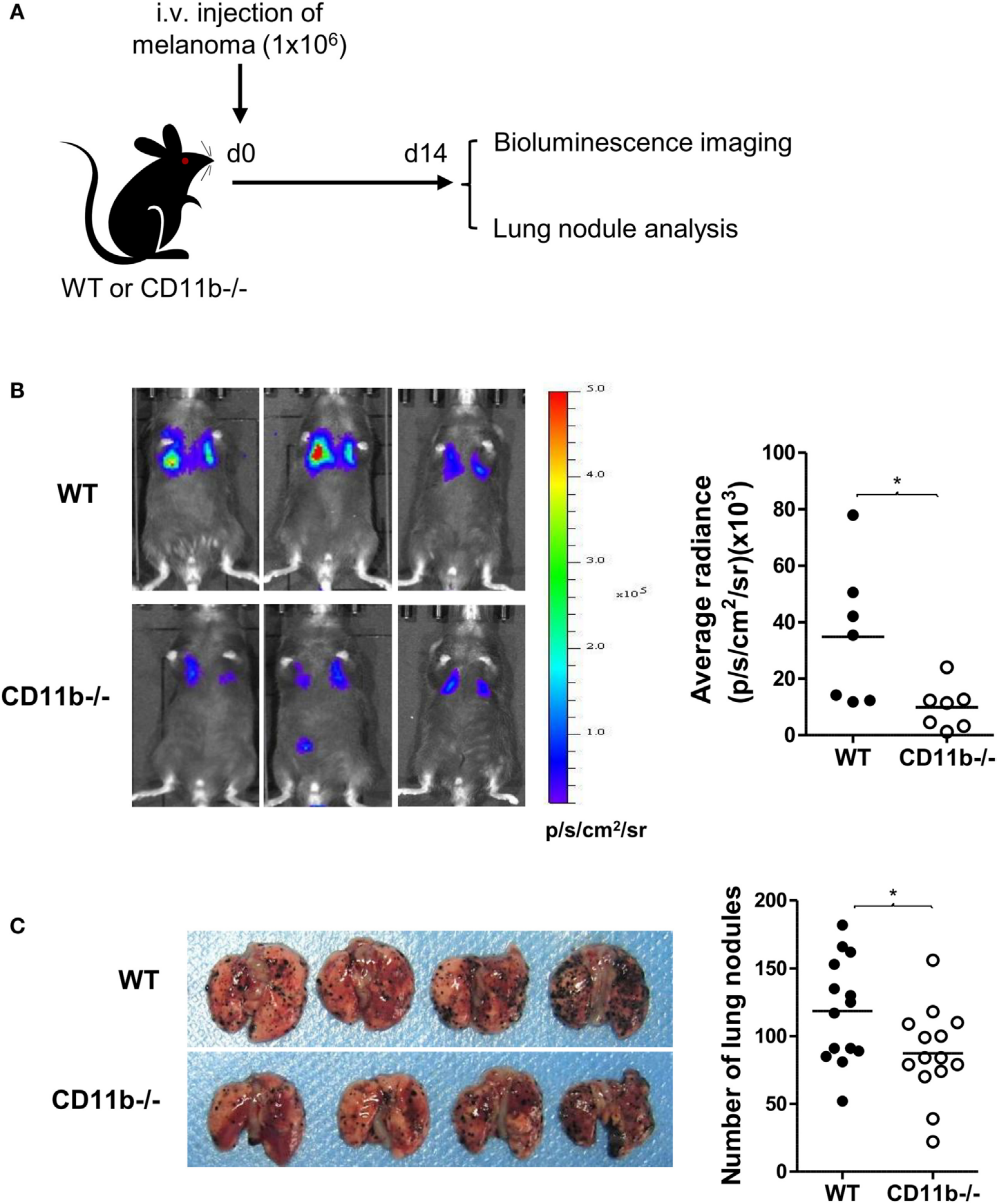
CD11b^−/−^ mice have reduced metastatic tumor burden. **(A)** Schematic diagram of experimental design. WT or CD11b^−/−^ mice received B16-luc melanoma cells by i.v. injection. Lung metastatic tumor burden was assessed at d 14 post tumor cell inoculation. **(B)** Bioluminescence imaging of B16-luc lung metastases. Left: representative bioluminescence images of WT and CD11^−/−^ mice. Right: quantification of average radiance (p/s/cm^2^/sr) from the two groups of mice (*n* = 7/group, pooled from two separate experiments), and data were analyzed by unpaired *t* test with Welch’s correction, *P < 0.05. **(C)** Lung nodule analysis (d 14). Left: representative images of lungs from WT and CD11b^−/−^ mice. Right: quantification of the number of tumor nodules on lung surface from the two groups of mice (*n* = 14/group, pooled from four separate experiments), and data were analyzed by unpaired two-tailed Student’s *t*-test.*P < 0.05. **(B,C)** Each dot represents an individual mouse; small horizontal lines indicate the mean.

**Figure 3:**
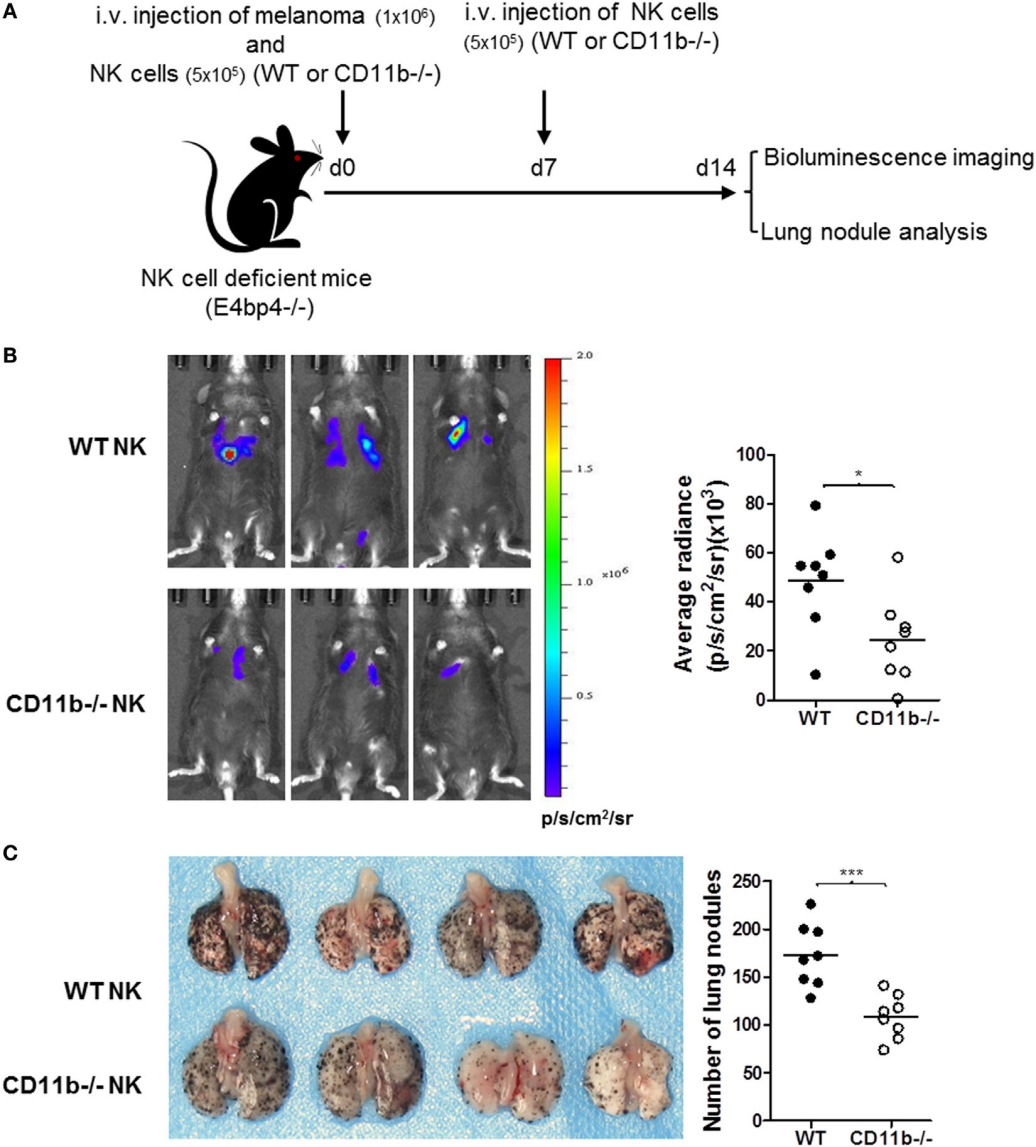
Mice that received CD11b^−/−^ natural killer (NK) cells have less lung metastatic tumor burden than mice that received WT NK cells. **(A)** Schematic diagram of experimental design. NK cell-deficient mice (E4bp4^−/−^) that received B16-luc melanoma cells and NK cells from WT or CD11b^−/−^ mice by i.v. injection at d 0, and received second dose of NK cells at d 7. Lung metastatic tumor burden was assessed at d 14 post tumor cell inoculation. **(B)** Bioluminescence imaging of B16-luc lung metastases. Left: representative bioluminescence images of mice received WT or CD11b^−/−^ NK cells. Right: quantification of average radiance (p/s/cm^2^/sr) from the two groups of mice (*n* = 8/group). **(C)** Lung nodule analysis. Left: representative images of lungs from the mice received WT or CD11b^−/−^ NK cells. Right: quantification of the number of tumor nodules on lung surface from the two groups of mice (*n* = 8/group). **(B,C)** The data were generated from two independent experiments and analyzed by unpaired two-tailed Student’s *t*-test.*P < 0.05. Each dot represents an individual mouse; small horizontal lines indicate the mean.

Given that NK cells play important roles in tumor surveillance (35), and NK cell functional activity is suppressed by CR3, we hypothesized that CR3-dependent suppression of NK cell function will have negative impact on tumor surveillance, and lack of CR3 will release the suppression and enhance NK cell-dependent tumor surveillance. To test this hypothesis, we employed a pulmonary B16 melanoma metastases model and assessed tumor burden in the lung of CD11b^−/−^ and WT mice. 14 days after intravenous inoculation of the melanoma cells, compared with WT mice, CD11b^−/−^ mice had significantly reduced metastatic tumor burden measured by bioluminescence imaging analysis and less lung nodules measured by visually scoring surface tumor nodules (Figures 2A–C). These results clearly demonstrate that CR3 has negative impact on tumor surveillance. We next assessed whether CR3-depedent suppression of NK cell function contributes to the impairment of tumor surveillance. We employed an adaptive transfer of NK cell approach in the abovementioned melanoma metastasis model, except the recipient mice were E4bp4^−/−^ mice (which had functional NK cell deficiency) (32). We initially examined the engraftment of NK cells in E4bp4^−/−^ mice. CR3-sufficient NK cells (from WT mice) and CR3-deficient NK cells (from CD11b^−/−^ mice) were similarly grafted in tumor bearing E4bp4^−/−^ mice 24 h after intravenous injection of the NK cells (Figure S5 in Supplementary Material), which suggest that CR3-deficient NK cells have no apparent defect in migration in this model. We then assessed the impact of the two groups of NK cells on tumor suppression. Following intravenous inoculation of the melanoma cells, mice that received two doses (d0, d7) of CR3-deficient (CD11b^−/−^) NK cells by intravenous injection had significantly reduced metastatic tumor burden and less lung nodules, compared with the mice that received CR3-sufficient (WT) NK cells (Figures 3A–C), exhibiting a similar level of reduction in tumor nodules and burden as observed in CD11b^−/−^ mice (Figures 2A–C). These findings support that CR3-mediated suppression of NK cell function has a negative impact on tumor surveillance in this model.

### iC3b-Containing Serum Has Negative Effects on NK Cell Activity and Function

**Figure 4:**
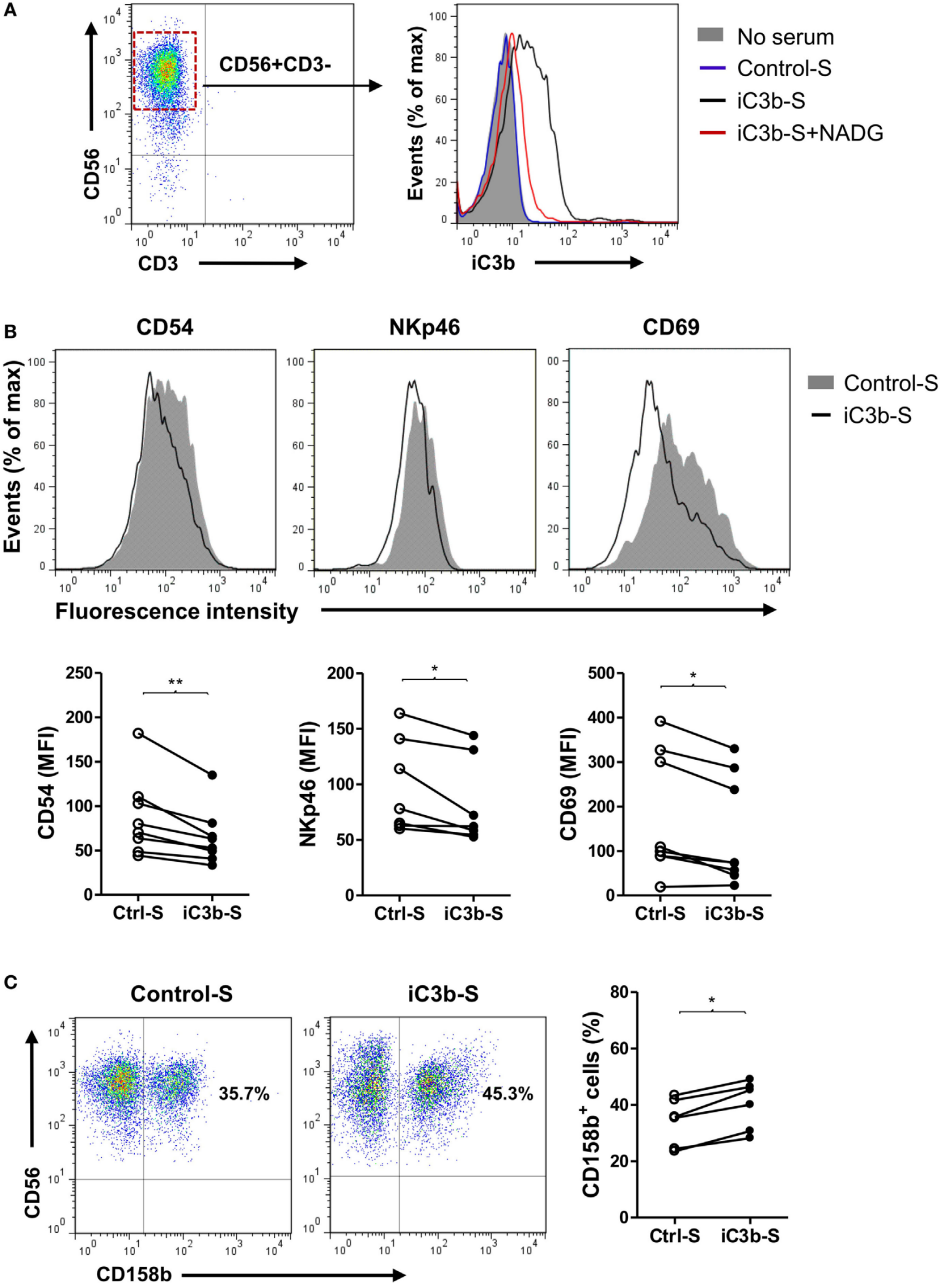

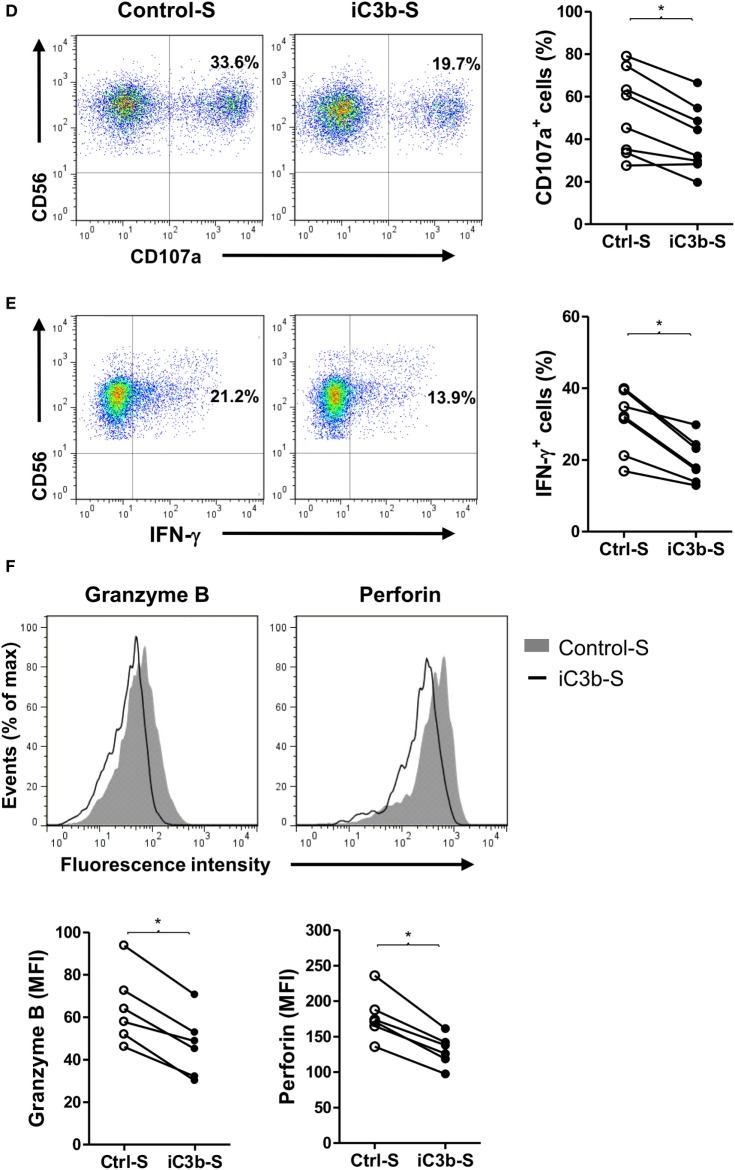
iC3b-containing serum has negative effects on natural killer (NK) cell activity and function. Purified human NK cells from healthy donors were incubated with or without 10% of the following sera [including zymosan-treated normal serum (iC3b-S), zymosan-treated C3-depleted serum (Control-S) or iC3b-S plus 50 mM *N*-acetyl-d-glucosamine (NADG)] for different time periods. **(A)** Flow cytometric analysis of deposition of iC3b on NK cells (1 h after the incubation) using mouse anti-human iC3b (neo) antibody. A representative of three independent experiments with three cell preparations is shown. **(B,C)** Flow cytometric analysis of surface expression of activating [CD54, NKp46, CD69, in **(B)**] and inhibitory (CD158b), in **(C)** receptors on NK cells (24 h after the incubation). **(D–F)** Flow cytometric analysis of expression of NK cell functional markers (CD107a, Granzyme B, and perforin) and production of IFN-γ in NK cells that had been incubated with serum for 24 h and further stimulated with K562 tumor cells. **(B–F)** The results were derived from six to eight experiments using different blood donors and analyzed by paired two-tailed Student’s *t*-test. *P < 0.05, **P < 0.01.

Next, we explored the mechanisms by which CR3 mediates the suppression of NK cell function. iC3b is present in the circulation under normal conditions and known to increase in certain pathological states including cancer (31), and soluble iC3b can bind to CR3 on cell surface (29). Therefore, we investigated the effects of soluble iC3b on NK cell activity and function using freshly prepared human NK cells and iC3b-containing serum. We first measured iC3b levels in normal human serum and iC3b-containing serum (zymosan-treated normal serum) and examined whether iC3b binds to NK cells. iC3b was detected in normal serum and zymosan-treated serum (91.2 ± 4.8 and 361.2 ± 23.5 μg/ml, respectively), but not detected in control serum (zymosan-treated C3-depleted serum). Flow cytometry analysis showed that bound iC3b was detected on NK cell surface, following incubation of NK cells with iC3b-containing serum for 1 h, but was absent when NK cells were incubated with control serum, and the binding was effectively blocked by preincubation of iC3b-containing serum with *N*-acetyl-d-glucosamine (NADG) (a naturally occurring sugar that prevents iC3b binding to CR3), indicating that soluble iC3b can bind to NK cells, and the binding is CR3 specific (Figure 4A). Next, we assessed the effects of iC3b-containing serum on NK cell activity and function. Expression of the activating receptors/markers CD54, NKp46, and CD69 was significantly reduced, whereas the inhibitory receptor CD158b was significantly increased on NK cells by incubation with iC3b-containing serum for 24 h, compared with control serum (Figures 4B,C). Accordingly, expression of NK cell functional markers (i.e., CD107a, IFN-γ, granzyme B, and perforin) was significantly reduced on NK cells by incubation with iC3b-containing serum, following the stimulation with K562 tumor cells (Figures 4D–F). Furthermore, the effect of iC3b-containing serum on NK cell activity and function was blocked by NADG (Figure S6 in Supplementary Material). Together, these findings demonstrate that soluble iC3b can bind to CR3 on NK cells and negatively regulate NK cell activity and function.

### Bound iC3b Is a Potent Negative Regulator of Human NK Cell Activity and Function

**Figure 5:**
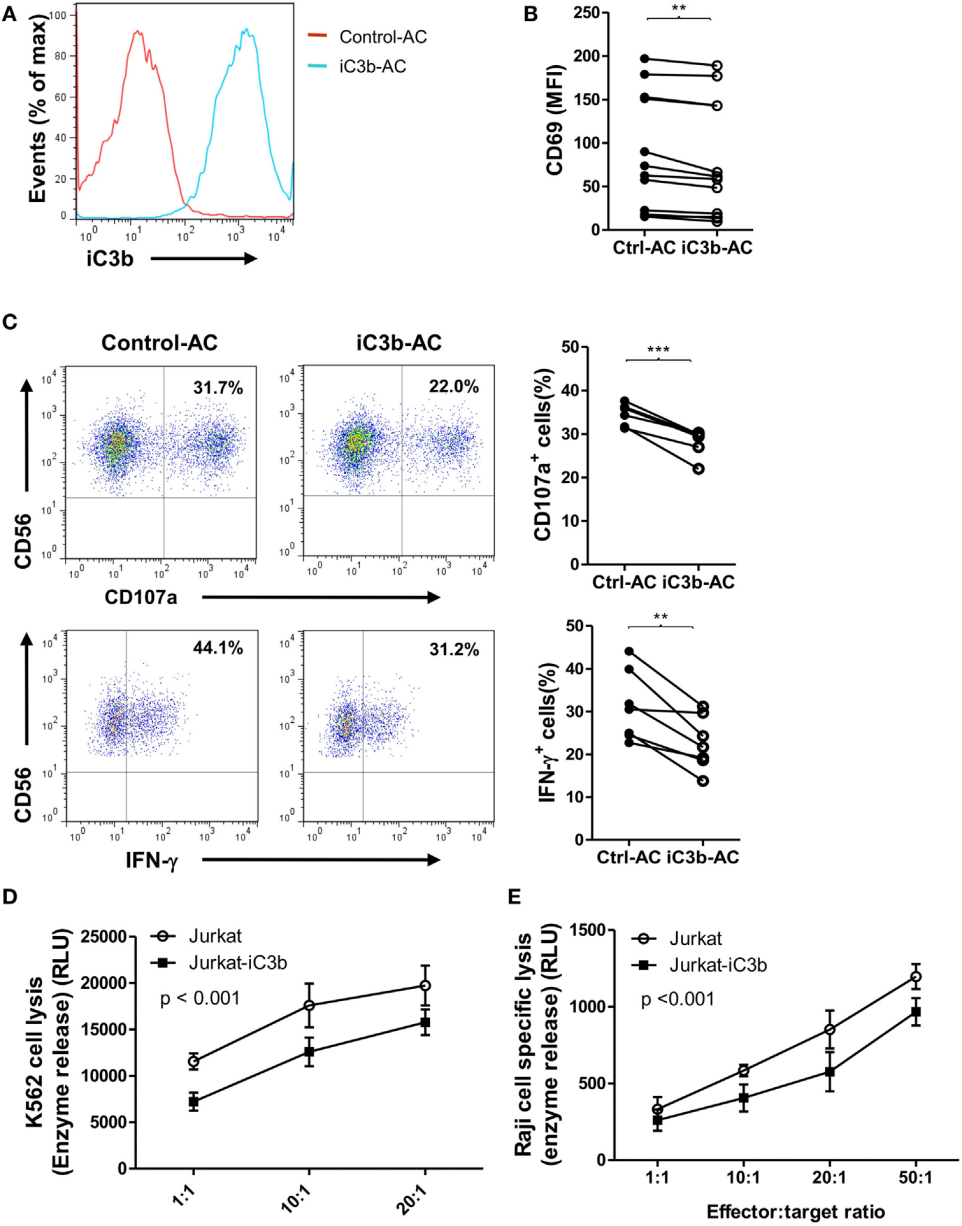
Bound iC3b is a potent negative regulator of natural killer (NK) cell activity and function. **(A)** Apoptotic Jurkat cells were incubated with either normal serum or C3-depleted serum for 1 h, and the deposition of iC3b on the surface was analyzed by flow cytometry using mouse anti-human iC3b (Neo) antibody. **(B–E)** Purified human NK cells from healthy donors were incubated with normal serum pretreated-apoptotic cells (iC3b-AC) or C3-depleted serum pretreated apoptotic cells (Control-AC) for 24 h and NK cell activity and function were assessed. **(B)** Flow cytometric analysis of surface expression of CD69 on gated CD56^+^CD3^−^ cells. **(C)** Expression of CD107a and production of IFN-γ in gated NK cells that had been further stimulated with K562 cells. **(D)** Direct killing of NK-sensitive K562 tumor cells by NK cells that had been pretreated with iC3b-AC or Control-AC. **(E)** Antibody-dependent cell-mediated cytotoxicity of rituximab-coated Raji cells assay was performed by using NK cells that had been pretreated with iC3b-AC or Control-AC. **(B,C)** Data were derived from 6 to 12 experiments using different blood donors and analyzed by paired two-tailed Student’s *t*-test. **P < 0.01, ***P < 0.001. **(D,E)** Data were generated from three independent experiments, with triplicate samples/group in each experiment and analyzed by two-way ANOVA.

Apoptotic cells and cancer cells often have iC3b deposited on their cell surface. Next, we investigated the effects of bound iC3b on NK cell activity and function using freshly prepared human NK cells and iC3b-apoptotic cells. We first confirmed the deposition of iC3b on apoptotic cells following incubation with normal serum (20%, for 1 h) (Figure 5A). Then, we cocultured NK cells with iC3b-apopototic cells or control cells (apoptotic cells preincubated with C3-depleted serum) for 24 h and assessed NK cell activity and function by flow cytometry. NK cells that had been cocultured with iC3b-apoptotic cells exhibited significantly lower levels of CD69 and functional markers (CD107a, IFN-γ) than that cocultured with control cells (Figures 5B,C). Furthermore, NK cells pre-cocultured with iC3b-apopototic cells induced less efficient killing of target cells, including the direct killing of NK-sensitive K562 tumor cells and ADCC of rituximab-coated Raji cells (Figures 5D,E). Collectively, these findings demonstrate that cell-bound iC3b can effectively suppress NK cell activity and cytotoxic function.

### Identification of Intracellular Signaling Pathways Responsible for the Action of iC3b on NK Cell Functional Regulation

**Figure 6:**
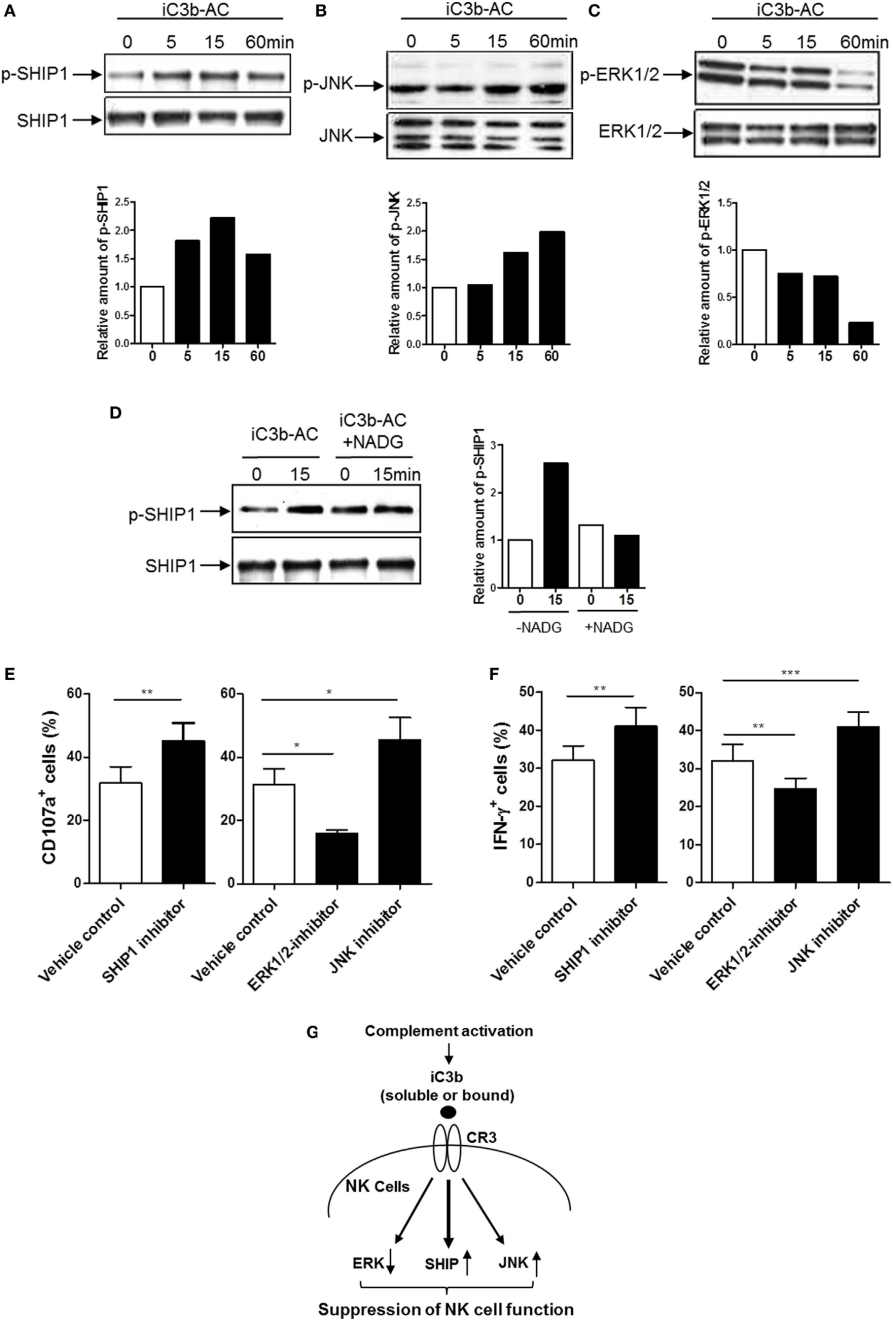
Identification of intracellular signaling pathways responsible for the action of iC3b on natural killer (NK) cell functional regulation. **(A–C)** Western blot for detection of phosphorylation of Src homology 2 domain-containing inositol-5-phosphatase-1 (SHIP1), ERK1/2, and JNK (MAPK) was performed in purified human NK cells that had been stimulated with bound iC3b (iC3b-AC) for indicated time periods. In each blot the top of bands corresponds to incubating membrane with appropriate phospho-antibody, and the bottom row of bands corresponds to incubating membrane with appropriate total antibody. Relative amounts of p-SHIP1, p-ERK1/2, and p-JNK are shown in the lower panel of the each figure. A representative of 3–4 independent experiments is shown. **(D)** Western blot for detection of SHIP1 was performed in purified human NK cells that had been stimulated with *N*-acetyl-d-glucosamine (NADG) pretreated iC3b-AC for indicated time periods. **(E,F)** Effect of inhibition of SHIP1, ERK1/2, and JNK pathways on CD107a expression and IFN-γ production in NK cells. Purified NK cells were further cultured for 16 h in the presence or absence of the appropriated inhibitor (i.e., 3-alpha-aminocholestane for SHIPI, U0126 for ERK, SP600125 for JNK) and the vehicle control then were used for functional assays. Results are presented as percentage of CD107a^+^ or IFN-γ^+^ cells in gated CD56^+^D3^−^ cells. Data were expressed as mean ± SEM of four independent experiments and analyzed by paired two-tailed Student’s *t*-test. **P < 0.01, ***P < 0.001. **(G)** Proposed intracellular signaling pathways responsible for the action of iC3b on NK cell functional suppression. iC3b stimulation mediates activation of SHIP and JNK and inhibition of ERK, which lead to suppression of NK cell function.

Having demonstrated that iC3b has negative effects on NK cell activity and effector function, we next sought to identify the signal transduction pathways through which iC3b may negatively affect NK cells. Binding of ligands to integrin (outside-in signaling) triggers a variety of intracellular signaling. Activation of the Src homology 2 domain-containing inositol-5-phosphatase-1 (SHIP-1) and downregulation of JNK and MAPK activation have been suggested as important signal transduction pathways involving negative regulation of cellular responses in different types of cells (e.g., macrophage, B cell, and NK cell) (36, 37). We hypothesized that CR3 may suppress NK cell function through the abovementioned intracellular pathways. We stimulated human NK cells with cell-bound iC3b and analyzed the changes of phosphorylation of SHIP-1, ERK, JNK, AKT, and P38 in NK cells. Phosphorylation of SHIP-1 and JNK were upregulated, in contrast, the ERK was downregulated by iC3b stimulation, as measured by both flow cytometry (Figure S7 in Supplementary Material) and Western blot (Figures 6A–C). No significant changes were observed in AKT and P38 phosphorylation following iC3b stimulation (Figure S8 in Supplementary Material). In addition, blockade of iC3b binding to CR3 by preincubation of iC3b with NADG significantly inhibited the effects of iC3b on SHIP-1 phosphorylation (Figure 6D). To assess whether these intracellular signaling pathways responsible for the action of iC3b on NK cell functional regulation, we pretreated human NK cells with specific inhibitor of SHIP-1 (3AC) (33), ERK (U0126) and JNK (SP600125) and then assessed NK cell functional activity by flow cytometry, percentage of CD107a^+^ NK cells or IFN-γ^+^ NK cells significantly increased by SHIP-1 or JNK inhibition, but decreased by ERK inhibition (Figures 6E,F). Collectively, Western blot and flow cytometry analyses reveal that iC3b stimulation causes the activation of SHIP-1 and JNK and suppression of ERK in human NK cells; pathway inhibition experiments indicate that SHIP-1, JNK, and ERK are important NK cell signaling pathways, supporting that iC3b mediates negative regulation on NK cell functions through activation of SHIP-1 and JNK and inhibition of ERK signaling.

## Discussion

Several lines of emerging evidence have challenged the traditional concept about the role of complement in tumor surveillance. Tumor cells were often thought to develop resistance to complement mediated killing by increasing the expression of complement regulatory proteins, which promotes the conversion of C3b (active) to iC3b or C3d (inactive), thus preventing C5b-9 formation (38). In this context, previous studies have shown that complement C3- or C5-deficient mice had reduced tumor growth; complement cleavage products (e.g., C5a) through engagement with their receptors on immune cells negatively regulate anti-tumor immunity and subsequently promote tumor growth or metastasis (39, 40). Furthermore, rituximab-mediated lymphoma killing did not benefit from the activation of complement (41, 42). These studies clearly suggest that complement activation can contribute to tumor growth and metastasis through different mechanisms. In the present study, we show that CR3 impairs tumor surveillance by suppression of NK cell function, which provides novel insight into dysregulation of tumor surveillance by complement.

Although iC3b/CR3 axis has been implicated in pancreatic carcinoma (29, 31), it is unknown whether CR3 can more specifically through its effects on NK cells influence tumor surveillance. To investigate this, we employed several well-established animal models to assess NK cell anti-tumor activity *in vivo* (34, 43) and a series of *in vitro* analyses to assess NK cell activity and tumor cell killing function. Using the pulmonary B16 melanoma metastases model, we found that CR3-deficient mice conferred protection against lung metastases, suggesting that CR3 plays a critical role in suppression of anti-tumor immunity in this model. Combining an adaptive transfer of NK cell approach to the melanoma metastases model, we found that CR3-deficient NK cells mediated better anti-tumor responses than CR3-sufficient NK cells, defining the role of CR3 in suppression of NK-dependent anti-tumor immunity. Using the NK-dependent peritoneal tumor elimination model, we found that CR3-deficient mice rejected lymphoma cells better than WT mice, further supporting the role of CR3 in NK cell functional suppression. Therefore, results obtained from our *in vivo* experiments suggest that CR3 has strong suppressive effects on NK cell function that impairs NK-mediated anti-tumor responses.

**Figure 7:**
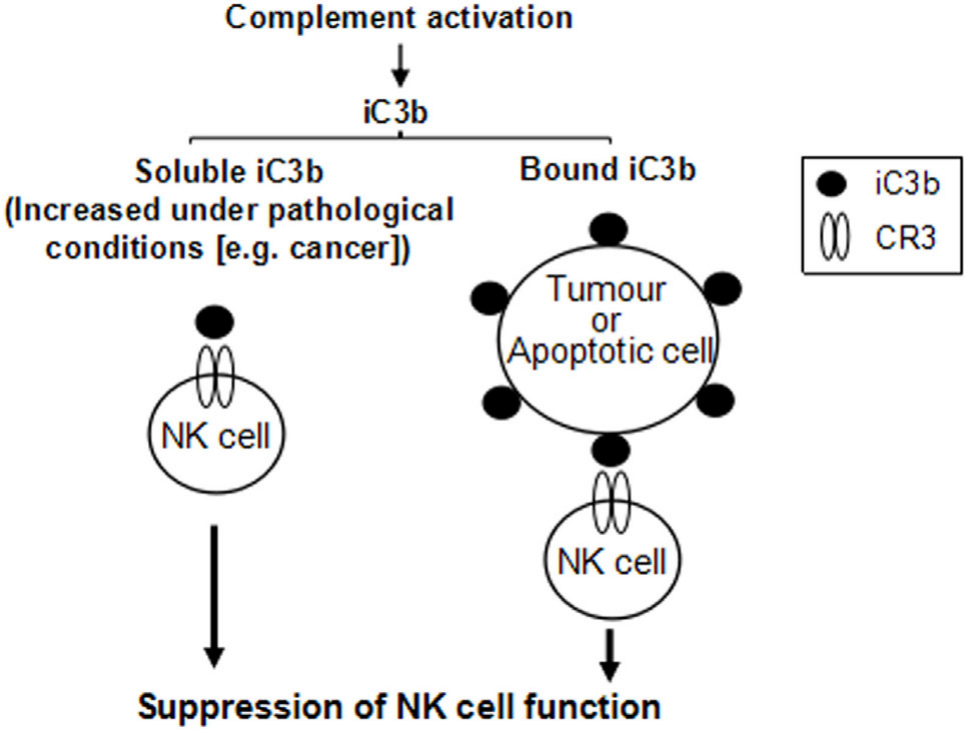
Schematic diagram illustrates that both soluble and cell-bound iC3b could mediate natural killer (NK) cell suppression. iC3b, either in soluble or cell-bound form, interacts with complement receptor 3 (CR3) expressed on NK cells, which induces suppression of NK cell function through receptor-mediated intracellular signaling pathways.

Our *in vitro* analyses found that both soluble and bound iC3b can suppress NK cell function through interaction with CR3. These observations for several reasons could have *in vivo* relevance. First, soluble iC3b is present in the circulation under normal conditions and increases in certain pathological states including cancer, as a result of increased complement activation (31). In cancer, the deposited iC3b could be shed from tumor cells to further increase iC3b levels in the circulation (29). Thus, although low levels of iC3b present in the circulation under normal conditions may only have minimal effects on NK cells, in the presence of tumor cells, elevated soluble iC3b may enhance its interaction with CR3 on NK cells which mediates NK cell suppression. In addition, tumor cells often have elevated iC3b on the cell surface as a result of upregulating complement regulator expression. Deposited iC3b on tumor and apoptotic cells may interact with CR3 on NK cells to mediate NK cell suppression. Therefore, soluble and bound iC3b, either in the circulation or within the tumor microenvironment, could mediate NK cell suppression, as illustrated in Figure 7.

In addition to the relevance to tumor surveillance, our results in ADCC analysis also suggest that iC3b/CR3-mediated NK cell suppression may have the implications in antibody-based cancer therapies. Traditionally, it is thought that complement plays important roles in antibody-mediated therapies through mediating complement-dependent cytotoxicity (CDC) and antibody-dependent NK cell cytotoxicity. However, for the CDC, there is no clear evidence that serum (more specifically, complement activity) is required for the effects of therapeutic antibodies, possibly reflecting the tumor cells developed strategy to prevent C5b-9 formation (38, 44). For ADCC, in contrast to enhancement of NK cell cytotoxicity suggested by early studies (44, 45), recent studies have shown that C3b fixation on target cells inhibited NK cell activation and impaired ADCC (41, 42). Therefore, these evidence, together with our observation that addition of iC3b impaired rituximab-dependent cytotoxicity by NK cells in ADCC assays, suggest that, in the context of failure to mediate tumor cell lysis, complement activation may have negative impact on antibody-based cancer therapies through iC3b/CR3-mediated NK cell suppression.

Another important aspect of our *in vitro* observations is identifying the signal transduction pathways through which iC3b negatively affects NK activation and function. SHIP (also known as SHIP1) is well characterized as a negative regulator of immune cells (36). It has been shown that SHIP-1 can be phosphorylated following activation of various membrane receptors (e.g., BCR, FcR, and TCR) (46, 47). SHIP can also regulate many other intracellular signaling such as AKT, JNK, and MARK through its catalytic activity or non-catalytic activity (37). SHIP has been suggested playing important roles in CD16-mediated downregulation of NK cell cytotoxicity and IFN-γ production (48, 49). A recent study, in mouse models of lymphoma and colon cancer, has shown that transient and pulsatile inhibition of SHIP1 increased both NK and T cell responsiveness and reduced the growth of hematological and solid tumors in mice. These findings further support a role for SHIP1 signaling in suppression of NK cell function and immunosuppression in cancer (50). However, it has been unknown whether SHIP involves CR3-mediated negative regulation of NK cell function. Our results clearly showed that iC3b stimulation caused an increase in the phosphorylation of SHIP, in agreement with this observation, it has been reported that SHIP was involved in integrin signaling in platelets (51). Therefore, given the roles of SHIP 1 in suppression of NK cell function and tumor immunity, our results strongly support the notion that SHIP signaling driven by iC3b/CR3 interactions in NK cells has negative impact on NK cell function and NK-dependent tumor surveillance. Our results also showed that increased SHIP phosphorylation was accompanied by a decrease in ERK1/2 phosphorylation, this is in agreement with the role of SHIP in inhibition of downstream signaling of MAPK as previously reported (52), and thus suggesting a SHIP-dependent ERK regulation. Although inhibition of JNK signaling by SHIP has been reported in other cells (e.g., B cells) (37), this was not observed in our study. On the other hand, we found that iC3b stimulation increased the phosphorylation of JNK in NK cells, suggesting that iC3b regulation of JNK is SHIP independent. In agreement with our observation, it has been suggested that CD11b (CR3) could involve the inhibition of NK cell function through activation of JNK pathway (53). Thus, our signaling experiments identified signaling transduction pathways responsible for iC3b/CR3-mediated NK cell suppression, namely activation of SHIP and JNK and suppression of ERK (Figure 6G).

In summary, the present study showed that CR3 impairs tumor surveillance by suppression of NK cell function and defined intracellular signaling pathways by which iC3b/CR3 interactions mediate NK cell suppression. Our findings provide new insight into the paradoxical effect of innate immune responses on tumor surveillance, such as complement activation and suggest that iC3b/CR3 axis is a potential therapeutic target for preserving NK cell function and improving NK cell-based therapy.

## Ethics Statement

This study was carried out in accordance with the recommendations of “School Human Studies Ethics Committee” with written informed consent from all subjects. All subjects gave written informed consent in accordance with the Declaration of Helsinki. The protocol was approved by the “School Human Studies Ethics Committee.” The animal experiment was carried out in accordance with the recommendations of “The Ethics Review Committee for Animal Experimentation at Xi’an Jiaotong University.” The protocol was approved by the “The Ethics Review Committee for Animal Experimentation at Xi’an Jiaotong University.”

## Author Contributions

CFL performed most in vivo experiments and participated in manuscript preparation. XYM performed most in vitro experiments. NW, JXW, NM, XD, BZ, and WW carried out some experiments. ZFL contributed to the interpretation of results. WZ and KL conceived and designed the research and wrote the manuscript. C-FL and KL analyzed data.

## Conflict of Interest Statement

The authors declare that the research was conducted in the absence of any commercial or financial relationships that could be construed as a potential conflict of interest.
