# Supplementary material for: Complement Receptor 3 Has Negative Impact on Tumor Surveillance through Suppression of Natural Killer Cell Function
Source: Front Immunol. 2017 Nov 20;8:1602. doi: 10.3389/fimmu.2017.01602 (PMC5702005; doi:10.3389/fimmu.2017.01602)
Supplement: Supplementary file 1 [file presentation_1.pdf]

sFigure 1. CD11b deficiency has no apparent effect on NK cell maturation

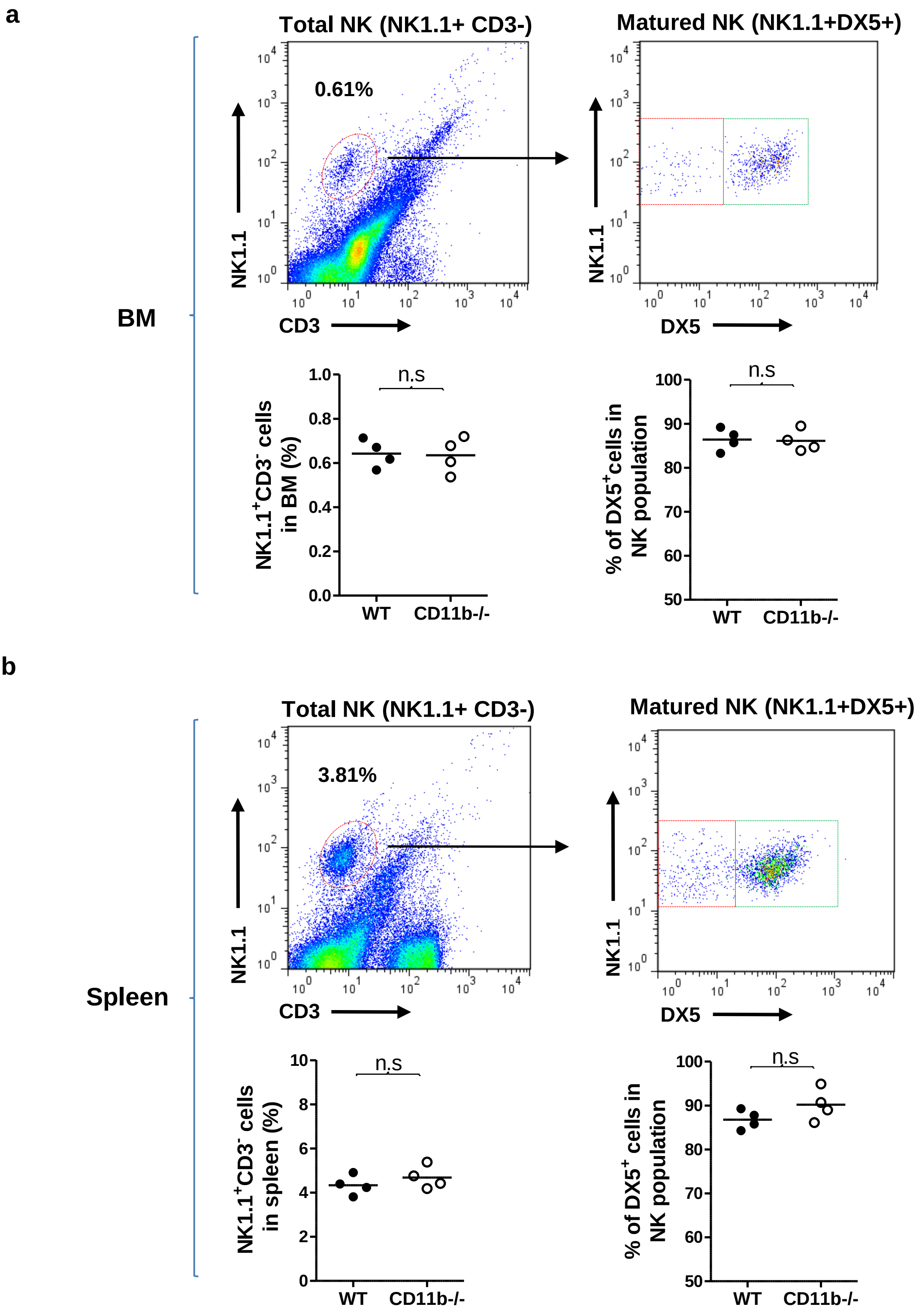

sFigure 1. CD11b deficiency has no apparent effect on NK cell maturation. Single population of bone marrow cells (**a**) and splenocyte (**b**) were prepared from WT and CD11b-/- mice and subjected to flow cytometry analysis for total NK cells (NK1.1+CD3-) and matured NK cells (CD3-NK1.1+DX5+). (**a, b**) Top panel: representative dot plot graphs of gated NK1.1+CD3- and CD3-NK1.1+DX5+ cells, lower panel: quantification of gated cells shown in the top panel. Data were analysed by Student's *t*-test, comparing CD11b-/- and WT mice. Each dot represents an individual mouse (n=4). Small horizontal lines indicate the mean.

sFigure 2. NK cells from CD11b-/- mice exhibit more activated phenotype

a

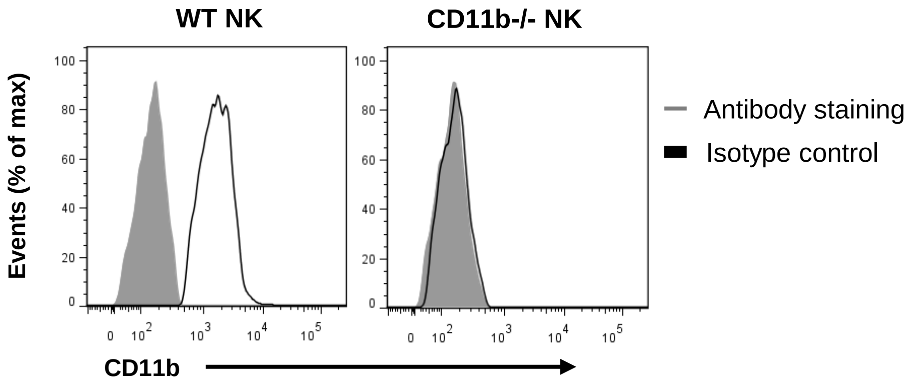

b

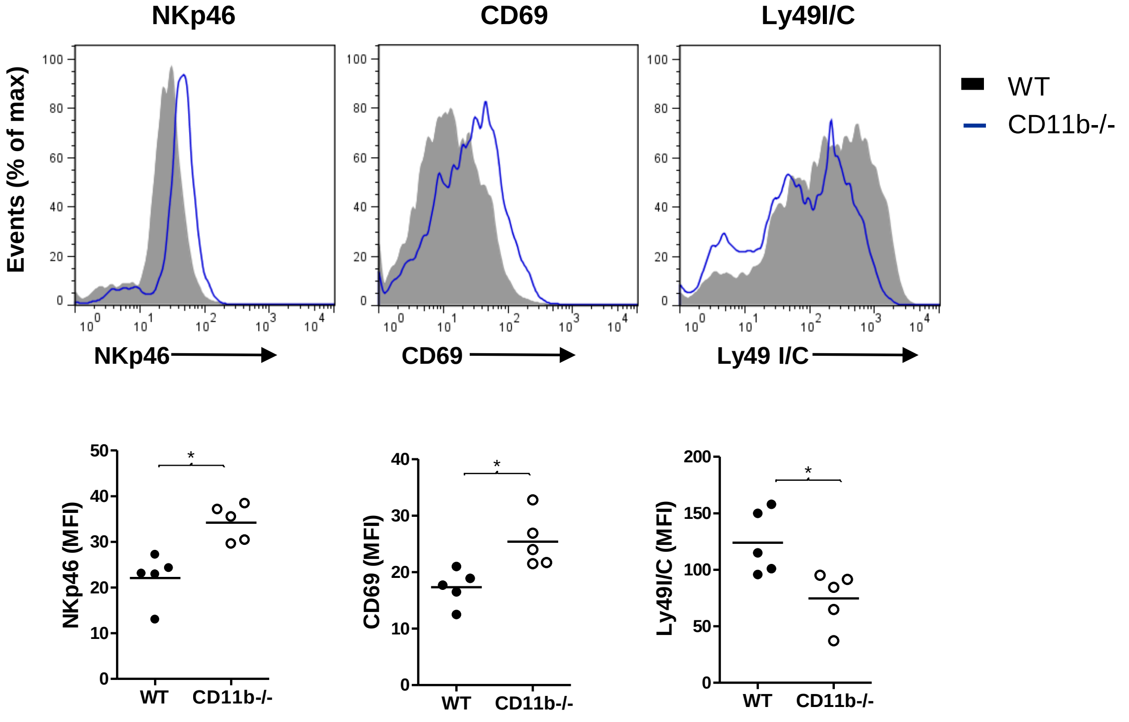

sFigure 2. NK cells from CD11b-/- mice exhibit more activated phenotype. Flow cytometry was performed in splenocytes from naïve WT and CD11b-/-mice. (**a**) CR3 (CD11b) was detected in NK cells ($NK1.1^{+}CD3^{-}$) from WT mice, but not in the NK cells from CD11b-/- mice. (**b**) NK surface phenotype markers (activating receptors NKp46 and CD69 and inhibitory receptor Ly49I/C) was determined in gated $NK1.1^{+}CD3^{-}$ cells. Top panel: representative histogram graphs for each molecules. Lower panel: quantified MFI. Data were analysed by Unpaired two-tailed Student's t test.* P<0.05. Each dot represents an individual mouse. Small horizontal lines indicate the mean.

sFigure 3. Peritoneal NK cells from CD11b-/- mice exhibit more activated phenotype

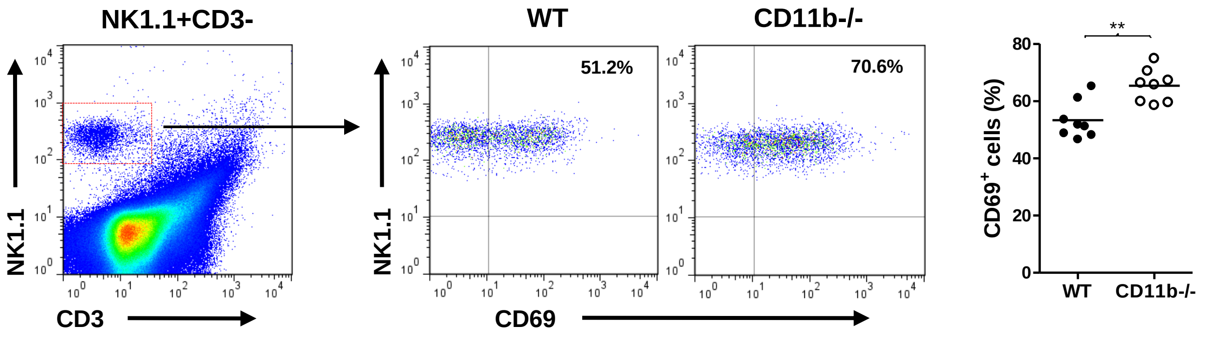

sFigure 3. Peritoneal NK cells from CD11b-/- mice exhibit more activated phenotype. 2.5×10^6 RMA-s cells were injected into the abdominal cavity of WT or CD11b-/- mice. 4h after injection, the peritoneal cells were collected and the expression of CD69 was determined in gated NK1.1$^+$CD3$^-$ cells. Left: representative flow cytometry dot plot, percentage of positively stained cells were indicated. Right: percentage of CD69+ cells obtained from 8 mice per group, data were analysed by Student's *t*-test, comparing CD11b-/- and WT mice. **P < 0.01. Each dot represents an individual mouse. Small horizontal lines indicate the mean.

sFigure 4. C3b/iC3b deposition on tumor cells

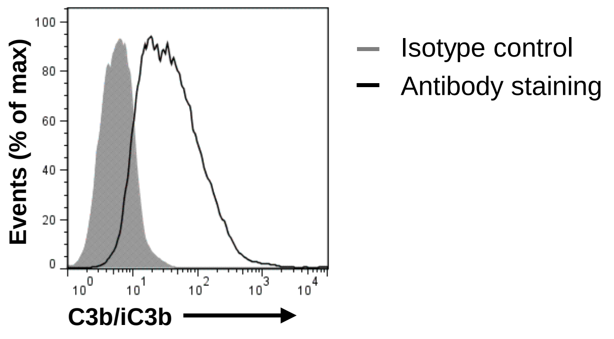

sFigure 4. C3b/iC3b deposition on tumor cells. CFSE labeled RMA-s cells (2.5x10^6) were injected into the abdominal cavity of WT or CD11b-/- mice. 1 hour after injection, the peritoneal cells were collected and stained with rat anti-mouse C3b/iC3b monoclonal Ab (3/26, Hycult Biotech). C3b/iC3b deposition on gated CFSE$^+$ cells was determined by flow cytometry. Data was shown as representative flow cytometry histogram from three independent experiments.

sFigure 5. CD11b deficiency has no apparent effect on NK cell migration

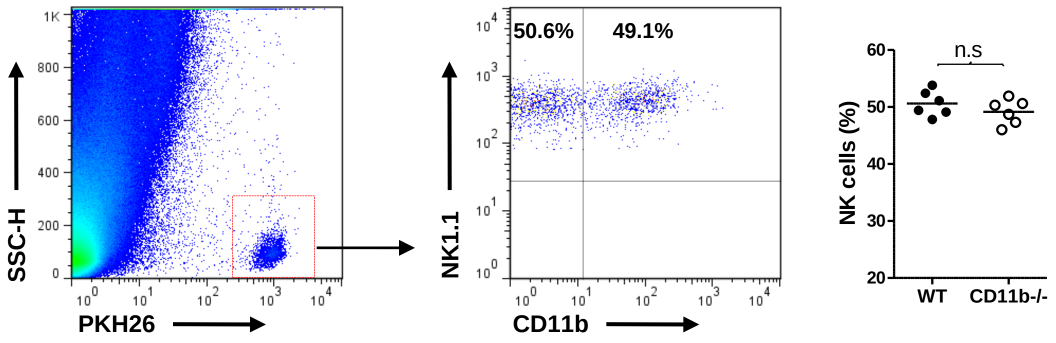

sFigure 5. CD11b deficiency has no apparent effect on NK cell migration. E4bp4-/- mice were inoculated with B16-luc cells ($1x10^6$) by i.v injection. At day 10, FACS-sorted NK1.1+CD3- NK cells from naïve WT and CD11b-/- mice were mixed (1:1) and labeled with PKH26, and injected into tumor-bearing E4bp4-/- mice. After 24h, lung tissues were harvested from the mice, the adoptive transferred NK cells were analysed by flow cytometry. Left: representative flow cytometry dot plots showing the adoptive transferred NK cells (in gated PKH26+cells) infiltration into lung tissues. Right: quantification of percentage of infiltrated WT and CD11b-/- NK cells. Experiments has been repeated in 6 mice, in two independent experiments.

sFigure 6. N-acetyl-D-glucosamine (NADG) treatment reduced the negative impact of iC3b-containing serum on NK cell function

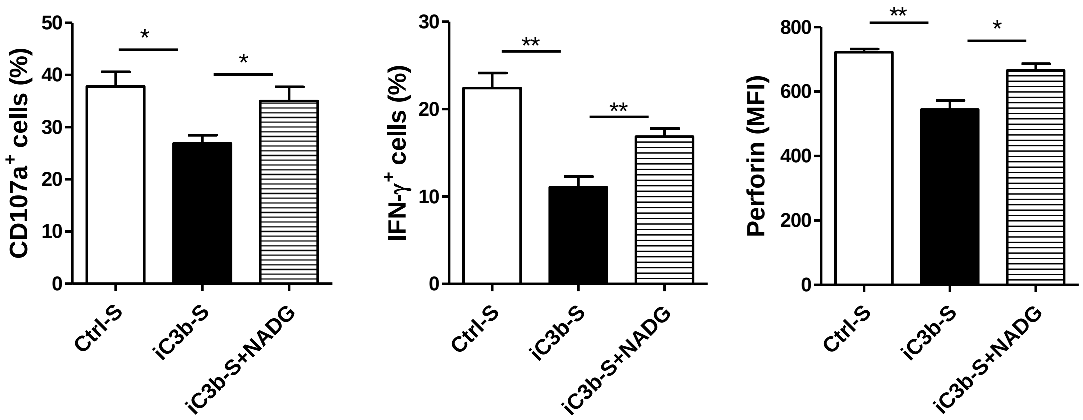

sFigure 6. N-acetyl-D-glucosamine (NADG) treatment reduced the negative impact of iC3b-containing serum on NK cell function. Purified human NK cells from healthy donors were cultured in the presence of 10% of serum for 5h (including zymosan-treated C3-depleted serum [Ctrl-S], zymosan-treated normal serum [iC3b-S], and iC3b-S plus 50mM NADG). NK cells were stimulated with K562 tumor cells and the expression levels of CD107a, IFN-γ and perforin were measured by flow cytometry. Collectively, results showed that iC3b-containing serum negatively regulates NK function, and in the presence of NADG can block the negative effect of iC3b-containing serum. The results were presented by mean fluorescence intensity (MFI) or percentage of positive cells (%). The result were pooled from 4 experiments using separate blood donors. Data were analysed by paired two-tailed Student's t test. * $P<0.05$,** $P<0.01$.

sFigure 7. Bound iC3b stimulation activates SHIP-1 and JNK but down regulates ERK signalling in NK cells

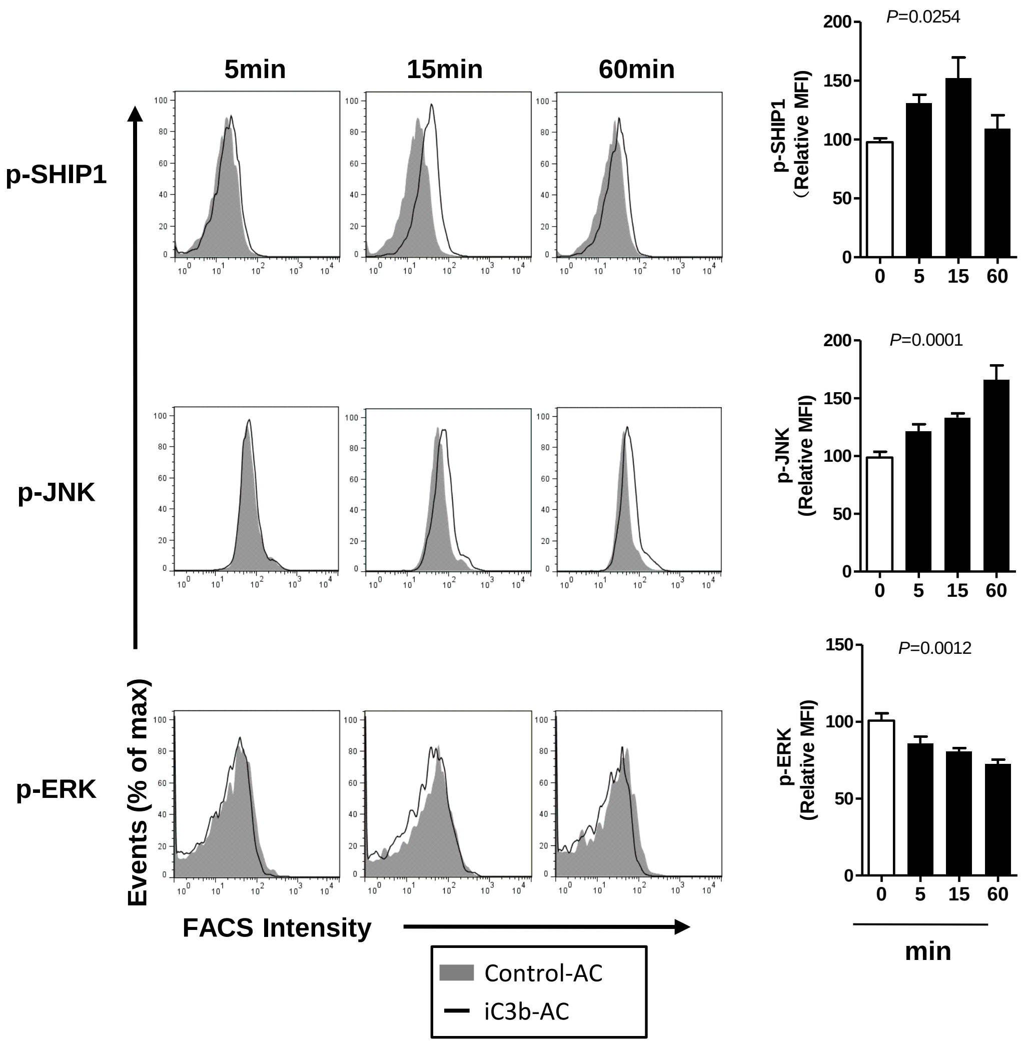

sFigure 7. Bound iC3b stimulation activates SHIP-1 and JNK but down regulates ERK signaling in NK cells. Purified human NK cells from healthy donors were incubated with normal serum pretreated-apoptotic cells (iC3b-AC) or C3-depleted serum pretreated apoptotic cells (Control-AC) at a 1:1 ratio for indicated time periods. The mixed cells were stained with anti-CD56 and anti-CD3 antibodies and permeabilised and stained with appropriate phospho-antibody. The phosphorylation of SHIP, JNK and ERK in gated $CD56^{+}CD3^{-}$ cells were analysed by flow cytometry. The left panel shows representative histogram plot overlay of the Control-AC treated NK (gray and black profiles) and iC3b-AC stimulated NK (open profiles). The right panel displays mean fluorescence intensity (MFI) of each phosphorylation protein. Data are presented as percentage of MFI of Control-AC stimulated NK cells at each time point, set as 100%. Data were expressed as means ± SEM of 3 independent experiments and analysed by one-way ANOVA.

sFigure 8. Bound iC3b stimulation has no notable effect on PI3K and P38 signalling in NK cells

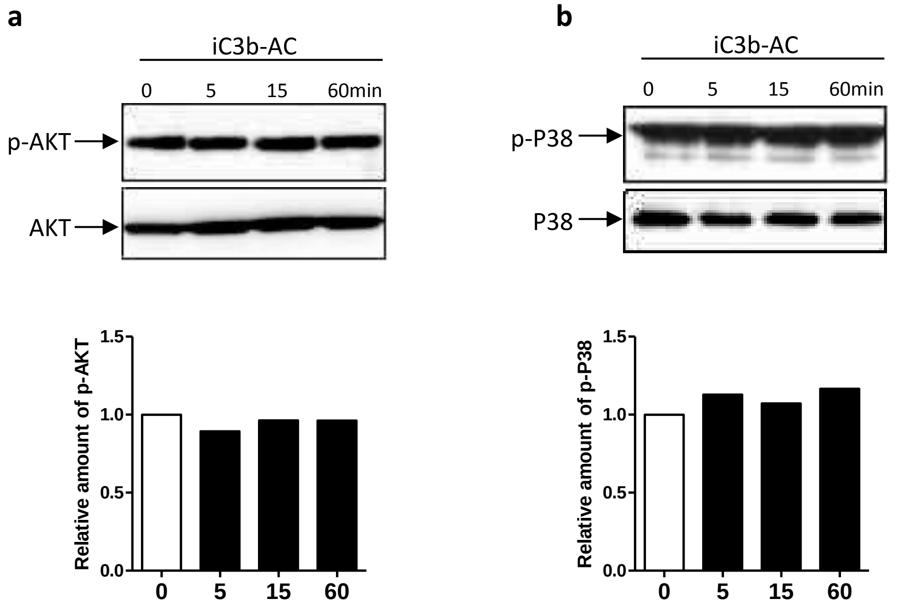

sFigure 8. iC3b stimulation has no notable effect on PI3K and P38 signaling in NK cells. Purified human NK cells from health donors were incubated with normal serum pretreated-apoptotic cells (iC3b-AC) at a 1:1 ratio for indicated time periods. The cell lysates were used for analyzing phosphorylation of AKT and P38 by Western blot. In each blot the top of bands corresponds to incubating membrane with appropriate phospho-antibody and the bottom row of bands corresponds to incubating membrane with appropriate total antibody. Relative amounts of p-AKT and p-P38 are shown in the lower panel of the each figure. A representative of 3-4 independent experiments is shown.
